# A Visual Color Response Test Paper for the Detection of Hydrogen Sulfide Gas in the Air

**DOI:** 10.3390/molecules28135044

**Published:** 2023-06-28

**Authors:** Hailong Zhang, Shiyu Li, Hongpeng Zheng, Zhongzhi Han, Bing Lin, Yingying Wang, Xiaojun Guo, Taigang Zhou, Haibing Zhang, Jianjun Wu, Hui Zhang, Junlei Tang

**Affiliations:** 1 School of Chemistry and Chemical Engineering & Institute for Carbon Neutrality, Southwest Petroleum University, Chengdu 610500, China; hailong0902@126.com (H.Z.); zhpcorrosion@163.com (H.Z.); h1900@foxmail.com (B.L.); tgzhou@swpu.edu.cn (T.Z.); 2 CNPC Engineering Technology Research Company Limited, Tianjin 300451, China; hanzz01@cnpc.com.cn (Z.H.); guoxj@cnpc.com.cn (X.G.); 3 Key Laboratory of Optoelectronic Chemical Materials and Devices (Ministry of Education), Jianghan University, Wuhan 430056, China; yingyingwanglyon@126.com; 4 State Key Laboratory for Marine Corrosion and Protection, Luoyang Ship Material Research Institute, Qingdao 266237, China; hbzhang20@hotmail.com; 5 Research Institute of Tianfu New Energy, Chengdu 610217, China; wumao-5@163.com

**Keywords:** H_2_S gas, colorimetric sensor, low detection limit, microleaks, visual monitoring

## Abstract

Hydrogen sulfide (H_2_S) is widely found in oil and natural gas wells and industrial wastewater tanks. Owing to its high toxicity, the monitoring and detection of H_2_S in the air is essential. However, recent techniques for the quantitative detection of H_2_S gas suffer from limitations such as high cost, complicated operation, and insufficient sensitivity, preventing their practical application in industry. Thus, we have developed a portable test paper for real-time and inexpensive monitoring of H_2_S gas by color changes. The test paper had a significantly low H_2_S detection limit of 200 ppb, which is considered safe for humans. Moreover, the color of the test paper did not change noticeably when exposed to CO_2_, N_2_, O_2_, and air environments, indicating that the test paper is selective for H_2_S gas and can be stored for a long time. In addition, we fitted a color difference linear model between the color difference values (Δ*E*) and the concentrations of H_2_S gas. The establishment of the linear model substantiates that the test paper can provide accurate intensity information when detecting H_2_S gas leakage.

## 1. Introduction

Hydrogen sulfide (H_2_S) is a suffocating, acidic, and highly toxic gas with a rotten egg odor that can be rapidly absorbed by the lungs [1]. H_2_S is mainly produced during the low-temperature coking of coal and refining of sulfur-containing oil and natural gas [2,3,4]. The decomposition of sulfur-containing amino acids in meat can also release a small amount of H_2_S gas [5]. Furthermore, sulfate-reducing bacteria in industrial wastewater can reduce sulfates to H_2_S under the action of organic matter, which not only contaminates drinking water but also corrodes pipes and equipment [6,7]. The U.S. Occupational Safety and Health Administration (OSHA) considers it safe for workers to be exposed to 10 ppm H_2_S for a maximum of 8 h. When the human body inhales H_2_S at concentrations greater than 5 ppm, olfactory nerve fatigue may occur, and inhalation of H_2_S concentrations beyond 50 ppm can cause convulsions or shock. Sudden death occurs when the human body inhales H_2_S at concentrations higher than 1000 ppm [8]. Therefore, judging a H_2_S gas leakage in the air by smelling could be dangerous, and it is necessary to detect H_2_S gas leakage through instruments.

Based on the reaction mechanisms, various methods have been developed to detect H_2_S gas, such as lead acetate-impregnated test papers, metal oxide semiconductor (MOS) materials, electrochemical sensors [9,10,11,12], and so on. The commercial technique for detecting H_2_S is lead acetate test paper. Although cheap and convenient, this method is less sensitive to H_2_S gas, with a limit of detection (LOD) between 5 and 10 ppm [13]. The electrochemical sensors are far more sensitive to H_2_S than lead acetate test paper. However, most commercially available sensors suffer from poor selectivity, pressure variation, limited temperature range, and short shelf life [14]. Similarly, H_2_S detection using MOS materials faces challenges such as high energy consumption, cross-sensitivity to other gases, and low stability [15]. The most significant disadvantage of MOS materials for industrial applications is their high cost. Some pH-sensitive materials can also visually indicate H_2_S gas [16,17], but they can be interfered with by other acid gases. Therefore, developing a multi-field, selective, portable, inexpensive, and stable method for H_2_S detection is essential.

On top of that, the application scenarios for sensors are also very limited. They can only detect H_2_S when site-directed, and cannot monitor the early leakage or microleakage of H_2_S, which is often the early form of large-scale H_2_S leakage events. If a H_2_S microleakage can be monitored in time, it can improve the safety of workers and reduce property damage. Unfortunately, there have been H_2_S leaks in recent years, such as a high-concentration hydrogen sulfide leak at a wastewater treatment plant in the Czech city of Plsen on 18 June 2021 [18]. On 17 April 2022, a H_2_S leak occurred in Yuyao, Zhejiang Province, China, resulting in the death of three employees after ineffective rescue [19]. Therefore, developing a multi-field, selective, portable, inexpensive, and stable method for H_2_S microleakage monitoring is essential.

Previously, our group found that anthocyanins had an obvious color response to H_2_S gas with a concentration of 10 ppm and a gradient response, indicating that the color change in the range of 10–1000 ppm was a linear function of the H_2_S concentration [20]. A test paper is a convenient, low-cost, and widely used detection method, such as an acid–base test paper and a biochemical test paper. In this study, anthocyanins and SbCl_3_ [21] were used as the H_2_S chromogenic reagents, and a filter paper was used as a carrier to prepare a simple, portable, accurate, and selective response test paper for gradient monitoring of the H_2_S. We confirmed that the H_2_S gas monitoring limit of the test paper was as low as 200 ppb and obtained the value of chromatism as a function of H_2_S concentration. We also studied the change in the microstructure of the test strip before and after exposure to the H_2_S environment and investigated the response mechanism of the test paper using SEM, XPS, and FT-IR analyses. In addition, we also found that SbCl_3_ not only reacts with H_2_S to produce Sb_2_S_3_, but also affects the reaction process of anthocyanins with H_2_S. Specifically, SbCl_3_ is adsorbed on the C=O of anthocyanins to form organic Sb_2_S_3_. This discovery proves that the test paper only undergoes characteristic reactions with hydrogen sulfide, reducing interference from other gases in the air.

## 2. Results and Discussion

The discussion in this chapter is based on three different types of test paper: SbCl_3_ test paper, anthocyanin test paper, and mixed test paper. First, we found that anthocyanin test paper was responsive to hydrogen sulfide gas, but there was a problem with its high detection limit. Next, we used a more stable inorganic reagent SbCl_3_ as a hydrogen sulfide indicator and found that although it lowered the detection limit, it was difficult to determine the leakage concentration of hydrogen sulfide. Finally, SbCl_3_ and anthocyanin were used as mixed indicators to successfully prepare a composite test paper with a concentration gradient response to hydrogen sulfide gas. The effects of hydrogen sulfide concentration, temperature, and interfering gas on the response performance of different test strips were studied. The working principle of the test paper was inferred through SEM, XPS, and other surface verification methods.

### 2.1. Factors Influencing Response Performance of Test Paper

#### 2.1.1. Indicator Concentration

**Figure 1:**
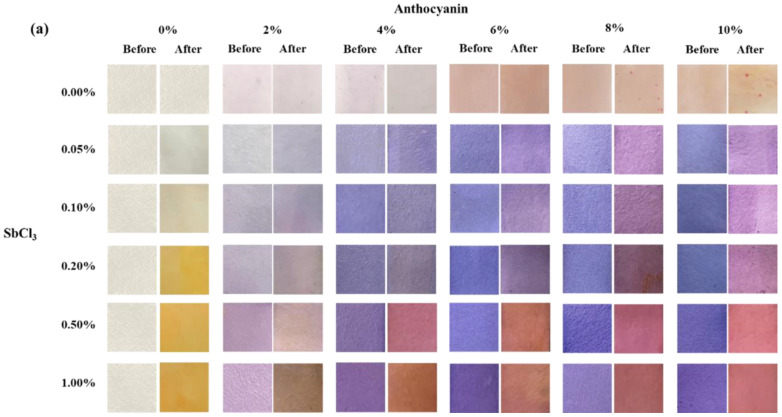

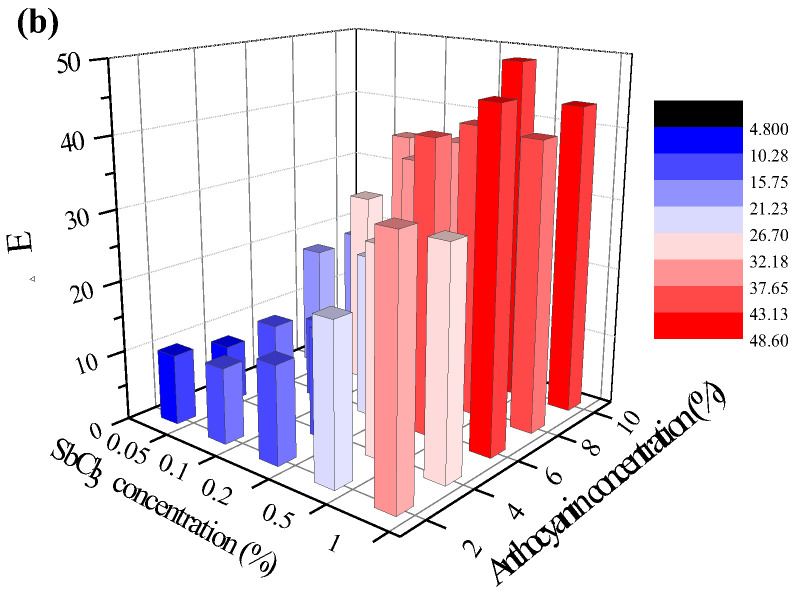
(**a**) Surface color change and (**b**) the color difference value ∆*E* of test paper with different indicator concentrations (SbCl_3_: 0.05, 0.1, 0.2, 0.5, 1.0 wt.%; anthocyanin: 2, 4, 6, 8, 10 wt.%) after being placed in 10 ppm H_2_S gas at 25 °C for 30 min.

The changes in the surface color and ∆*E* values of different test papers (coated with different concentrations of SbCl_3_ and/or anthocyanin) placed in 10 ppm H_2_S gas for 30 min are shown in Figure 1. As shown in Figure 1a, before the experiment, increasing anthocyanin concentration resulted in a gradual deepening of the blue on the test paper. However, the increase in SbCl_3_ concentration led to the gradual deepening of purple. For example, when the SbCl_3_ concentration was fixed at 0.05 wt.%, an increase in anthocyanin concentration led to a gradual change in the surface color of the test paper from light blue to dark blue, whereas at an anthocyanin concentration of 2 wt.%, the surface color of the test paper gradually turned from light blue to purple with the increase in SbCl_3_ concentration.

After exposure to 10 ppm H_2_S gas for 30 min, the surface of the test papers changed color to varying degrees. The surface of the anthocyanin test paper appeared red-dotted, while the SbCl_3_ test paper turned bright yellow due to an increase in indicator concentration. In the case of test papers coated with mixed solutions, an increase in anthocyanin concentration led to a gradual deepening of the brown on the test paper after the experiment. In contrast, an increase in SbCl_3_ concentration led to the gradual deepening of red. For example, after H_2_S exposure, the surface of the test paper coated with 1 wt.% SbCl_3_ and 2 wt.% anthocyanin turned brown while the surface of the test paper coated with 1 wt.% SbCl_3_ and 10 wt.% anthocyanin turned red.

Figure 1b shows the ∆*E* value digitized using a colorimeter, where a higher ∆*E* indicates a higher sensitivity of the test paper to H_2_S gas. ∆*E* gradually increased as the concentration of the indicator increased, reaching the maximum (∆*E* = 48.5) when the indicator was 0.5 wt.% SbCl_3_ and 10 wt.% anthocyanin. However, when the indicator was 0.5 wt.% SbCl_3_ and 4 wt.% anthocyanin, the test paper demonstrated a clear detection of H_2_S, and the ∆*E* did not increase significantly with further increase in the indicator concentration. Thus, the response test paper coated with 0.5 wt.% SbCl_3_ and 4 wt.% anthocyanins was selected for further testing and analysis.

#### 2.1.2. Temperature and Other Gases

**Figure 2:**
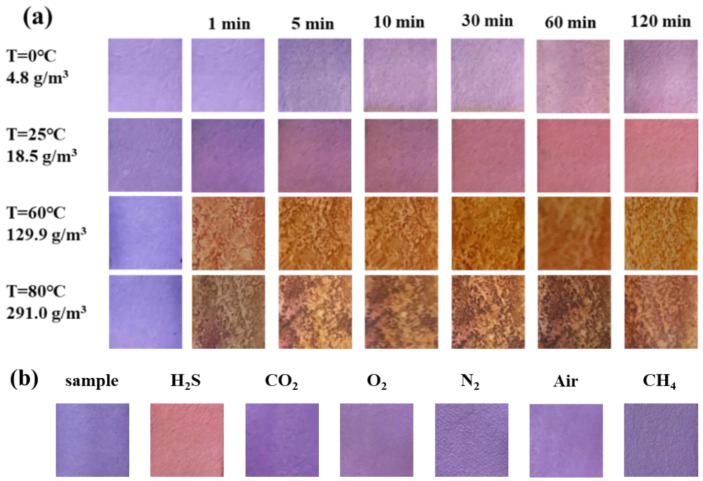
Change in surface color of test paper containing 0.5 wt.% SbCl_3_ and 4 wt.% anthocyanin (**a**) in 10 ppm H_2_S at different temperatures (0 °C, 25 °C, 60 °C, 80 °C) for 120 min, (**b**) in different gases (H_2_S, CO_2_, O_2_, N_2_, and air) at 25 °C for 120 min.

Figure 2a shows the color change in the test paper coated with 0.5 wt.% SbCl_3_ and 4 wt.% anthocyanin after exposure to 10 ppm H_2_S gas for 120 min at different temperatures. At 0 °C, the surface color of the test paper gradually changed from blue-violet to pink-purple within 30 min, while at 25 °C the blue-violet color on the surface quickly disappeared and turned pink. At 60 °C, the surface was entirely covered by yellow-brown color within 1 min, and at 80 °C the surface color rapidly turned black-brown.

Figure 2b shows the color change in the response test paper exposed to 10 ppm H_2_S and common gases (CO_2_, O_2_, N_2_, and air) for 120 min. The surface color of the test paper remained unchanged after exposure to a saturated CO_2_ environment for 120 min, indicating that CO_2_ did not interfere with the test paper. Similarly, the surface color did not change when exposed to O_2_, N_2_, and air, implying that test paper can be stored at room temperature for a long time without deterioration. The results indicate that the test paper has a characteristic response to H_2_S gas. Moreover, its low sensitivity to O_2_, N_2_, CO_2_, CH_4_, and air makes it a new method for the on-site detection of H_2_S gas.

#### 2.1.3. The Concentration of H_2_S Gas

**Figure 3:**
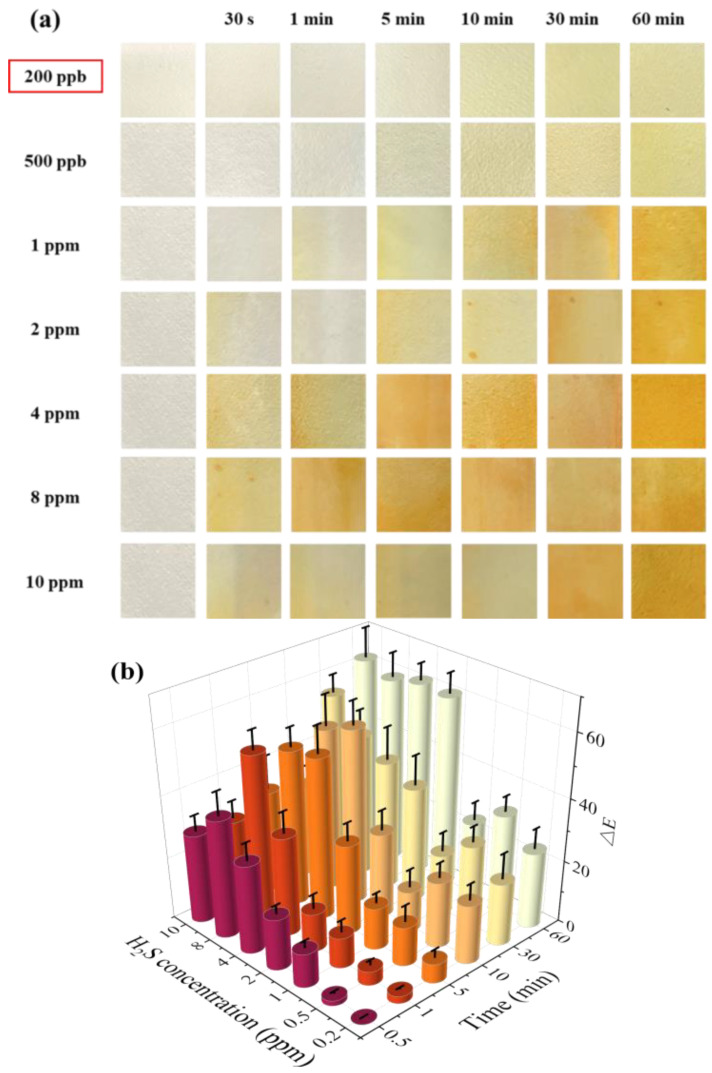
(**a**) Surface color change in 1 wt.% SbCl_3_ test paper in different H_2_S gas concentrations (200 ppb to 10 ppm) at 25 °C at different times (0 min to 60 min), (**b**) color difference value Δ*E*, different colors represent different exposure times.

Figure 3a shows the color change in the test paper coated with 1 wt.% SbCl_3_ after exposure to different H_2_S gas concentrations for different time intervals. The surface color of the test paper was white before the exposure, which turned into a uniform pale yellow within 10 min when exposed to H_2_S concentrations of 200 ppb and 500 ppb. However, at a higher H_2_S gas concentration of 1 ppm, the surface color changed quickly from white to pale yellow within 1 min, and the yellow gradually deepened with time. Further increase in the H_2_S gas concentration led to a faster response rate, with the surface color changing from white to yellow in 30 s at 4 ppm H_2_S. The above findings indicate that the test paper with 1 wt.% SbCl_3_ coating can quickly and efficiently detect H_2_S gas in the air with a detection limit as low as 200 ppb.

A colorimeter was used to extract the ∆*E* values of the test papers, and the color change was digitized using Equation (4). When the test paper coated with 1 wt.% SbCl_3_ was exposed to 10 ppm H_2_S for 60 min, the Δ*E* reached a maximum value of 62.26, as shown in Figure 3b. Moreover, the Δ*E* was 6.31 when the test paper was placed in a 200 ppb H_2_S for 10 min. Typically, Δ*E* > 3.3 is considered a critical value for determining whether the difference between the two colors can be observed through the naked eye [22]. However, similar Δ*E* values and faster reaction speed result in no obvious gradient response to varying concentrations of H_2_S gas using the test paper coated only with SbCl_3_, which makes it difficult to determine the concentration of H_2_S gas.

**Figure 4:**
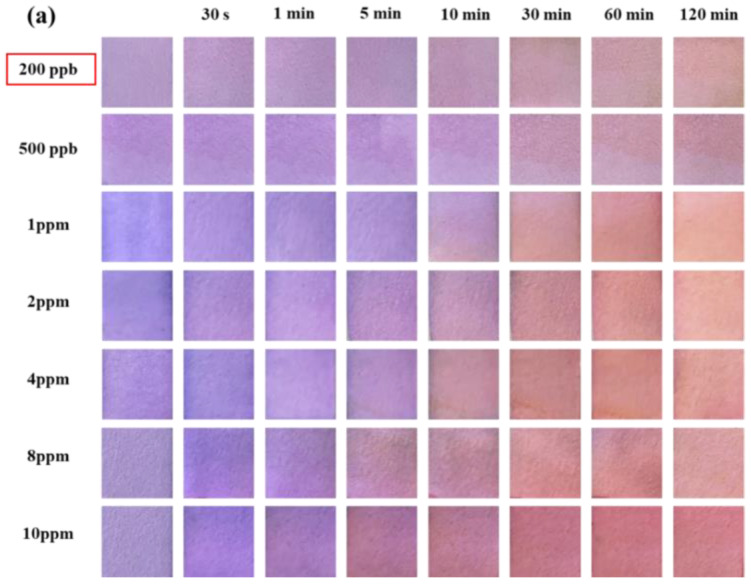

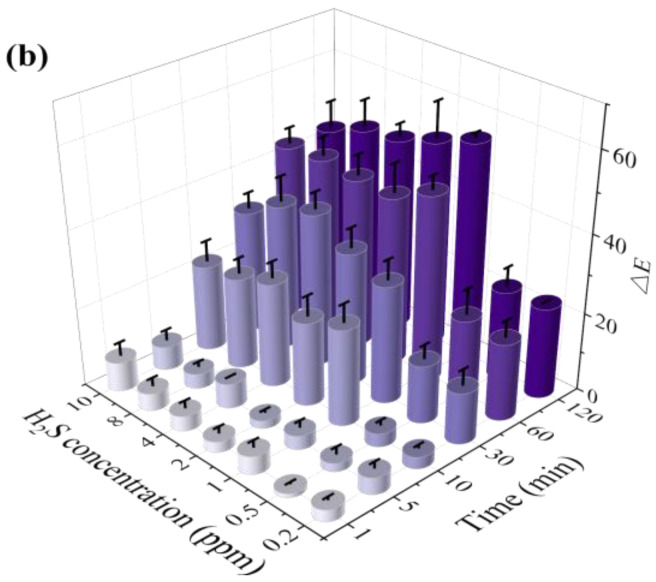
(**a**) Surface color change in 0.5 wt.% SbCl_3_ and 4 wt.% anthocyanin test paper in different H_2_S concentrations (200 ppb to 10 ppm) at 25 °C at different times (0 min to 120 min), (**b**) color difference value Δ*E*, different colors represent different exposure times.

Figure 4a shows the color change in the test paper coated with 0.5 wt.% SbCl_3_ and 4 wt.% anthocyanin after exposure to different H_2_S gas concentrations for different time intervals. The purple-blue surface color of the test paper turned into a uniform pale pink within 30–60 min when exposed to H_2_S concentrations of 200 ppb and 500 ppb. Furthermore, after exposure to 1 ppm H_2_S gas, the surface color of the test paper gradually turned purple to pink within 10–30 min. Further increase in the H_2_S gas concentration led to a faster response rate, with the test paper surface showing a uniform pink color in 5 min at 8 ppm and 10 ppm H_2_S concentrations. Thus, the H_2_S gas concentration significantly affects the color change rate of the test paper.

In the previous study, the detection limit of the response film containing only anthocyanin was 10 ppm [20]. In contrast, the composite test paper (coated with 0.5 wt.% SbCl_3_ and 4 wt.% anthocyanin) developed in this work demonstrated a significantly lower H_2_S detection limit of 200 ppb with a faster response rate. Compared to the response film, the surface color changes in the test paper were more uniform and stable. Moreover, the response film in the previous study was blue, and the composite test paper was blue-purple, suggesting the interaction of SbCl_3_ anthocyanin.

As shown in Figure 4b, after exposing the composite test paper to 1 ppm H_2_S for 60 min, the Δ*E* reached a maximum value of 55.55. However, the values of Δ*E* were 13.22 and 14.89 after 30 min of exposure to 200 ppb and 500 ppb H_2_S gas, respectively. Although the response time of the composite test paper was higher than the SbCl_3_ test paper, the surface color change in the composite test paper after 30 min was prominent to the naked eye. This indicated a lower H_2_S detection limit (200 ppb) of the composite test paper.

**Figure 5:**
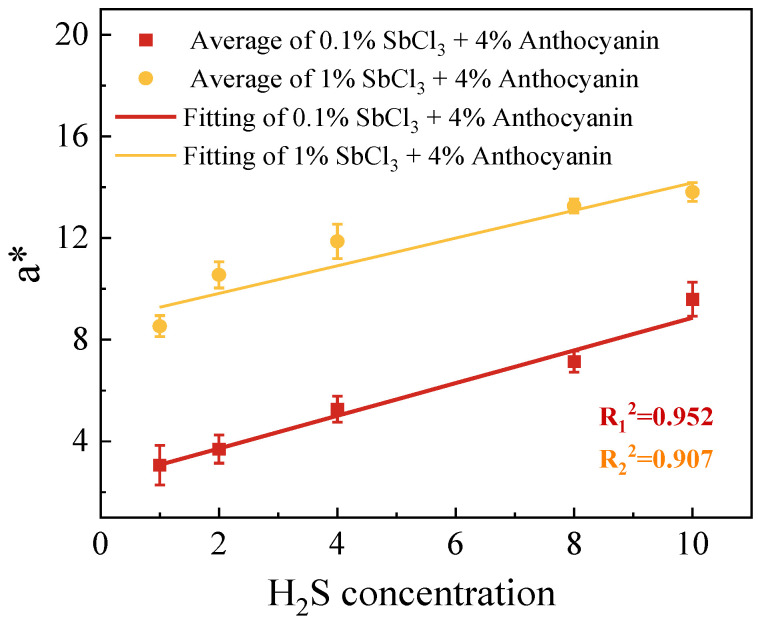
Linear fitting of a* of the surface color change in 0.5 wt.% SbCl_3_ and 4 wt.% anthocyanin test paper exposed to H_2_S gas for 30 min.

Although the SbCl_3_ and anthocyanin composite test paper had the disadvantages of a higher detection limit and slower detection speed than the 1 wt.% SbCl_3_ test paper, the Δ*E* and H_2_S concentration (1–10 ppm) showed a better gradient response (Figure 3 and Figure 4). As shown in Figure 5, we fitted a color difference linear model of the test paper coated with different concentrations of SbCl_3_ and 4 wt.% anthocyanins as a function of H_2_S concentration. We found a linear relationship between the values of Δ*E* and the concentrations of H_2_S in the range of 1–10 ppm. The Δ*E* from two test papers increased as the H_2_S concentration increased. The test paper with 0.5 wt.% SbCl_3_ and 4 wt.% anthocyanin coating showed a significant Δ*E* value and conformed to the linear model. Establishing the linear model provides a judgment standard for the practical application of the response test paper combined with an intelligent device and provides accurate intensity information when detecting H_2_S gas leakage. It is expected that it will be possible to recognize and digitize the color change in test paper with the future development of AI robots, and implement an automatic alarm when the H_2_S concentration reaches the dangerous range.

**Figure 6:**
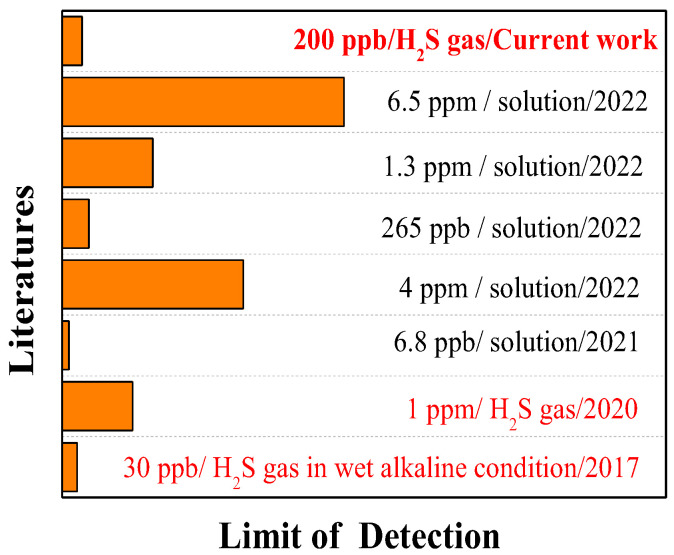
Research status of the detection limit of H_2_S visual sensor.

Figure 6 shows some excellent studies on the visual inspection of H_2_S. For example, the nanofibers prepared by Dong-Ha Kim [22] can detect H_2_S gas under dry conditions with a ppm-level detection limit (1 ppm). Compared with the gas phase, H_2_S detection in a solution is easier. For example, the fluorescent test paper prepared by Yan Feng [7] for H_2_S detection in wastewater had a low detection limit of 6.8 ppb. Similarly, the detection limit of the sensor designed by Yisheng Lin [23] for H_2_S detection in a solution was as low as 265 ppb. It is worth mentioning that the sensor prepared by Thomas S. [1] could detect 30 ppb of H_2_S gas, but it requires wet and alkaline conditions to achieve ppb-level detection. However, the H_2_S detection media developed by Xiaokun Yang [24], Xue Han [25], and Huanling Wu [26] had relatively higher H_2_S detection (in solutions) limits and the corresponding detection times were not clear. The response test paper developed in the current work can detect 200 ppb of H_2_S gas in a dry neutral environment without interference from other common gases and provide an accurate response time. Of course, the detection in wet conditions or detection methods based on nanomaterials may be our learnable methods for further lowering the H_2_S detection limit.

### 2.2. XPS Analysis of Test Paper

**Figure 7:**
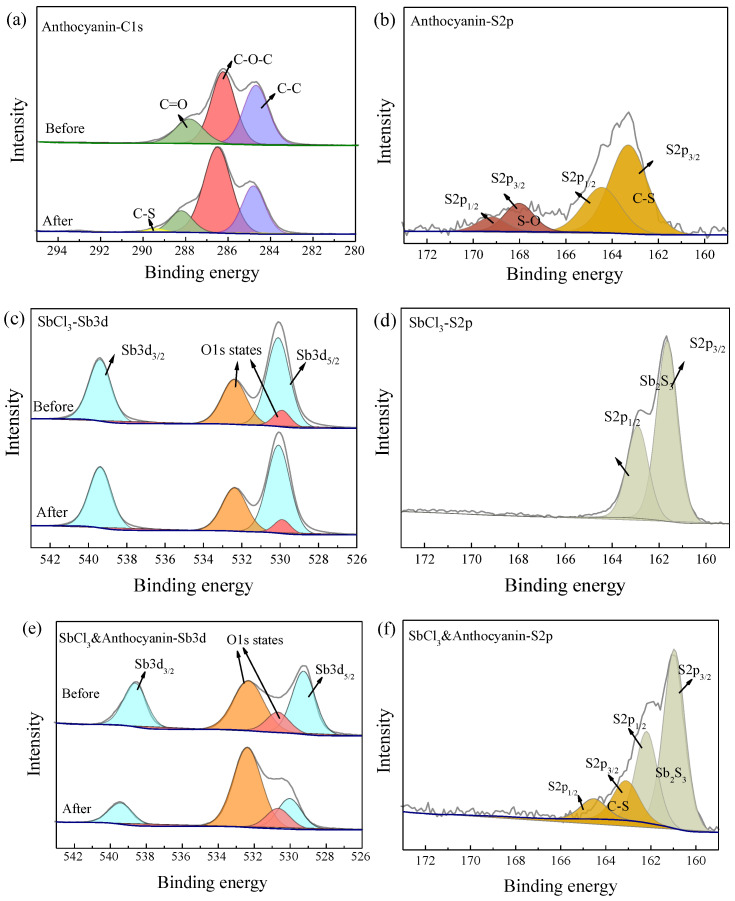
XPS spectra of three test papers before and after exposure to 10 ppm H_2_S at 25 °C in 30 min, peaks of different colors in the figure represent elements of different valence states: (**a**) C1s spectra of 4 wt.% anthocyanin, (**b**) S2p spectra of 4 wt.% anthocyanin, (**c**) Sb3d spectra of 0.5 wt.% SbCl_3_, (**d**) S2p spectra of 0.5 wt.% SbCl_3_, (**e**) Sb3d spectra of 0.5 wt.% SbCl_3_ and 4 wt.% anthocyanin, (**f**) S2p spectra of 0.5 wt.% SbCl_3_ and 4 wt.% anthocyanin.

To confirm the reaction mechanism between H_2_S and the response test paper, XPS was used to analyze the changes in the valence states of surface elements before and after exposure to 10 ppm H_2_S gas for 30 min. Figure 7a shows the binding energy peaks of 288.0 eV, 286.3 eV, and 284.8 eV corresponding to C=O, C-O-C, and C-C functional groups. The newly added binding energy peak of 289.4 eV after exposure to H_2_S corresponds to the C-S bond [27]. In the exposed S2p spectra (Figure 7b), binding energy peaks of 168.0 eV and 163.3 eV correspond to the S-O and C-S functional groups. This demonstrates a chemical reaction between anthocyanin test paper and H_2_S, which is consistent with the previous research findings [20].

As shown in Figure 7c, the binding energy peaks of the Sb element before and after the exposure of the 0.5 wt.% SbCl_3_-coated test papers were 539.3 eV and 529.9 eV, respectively, indicating that the Sb element always existed in the form of Sb (III) [28]. The exposed test paper showed binding energy peaks of 161.6 eV and 162.8 eV, indicating that SbCl_3_ and H_2_S reacted to form Sb_2_S_3_ [29].

Figure 7e shows that the binding energy peak shifts of the Sb element before the exposure of the composite (0.5 wt.% SbCl_3_ and 4 wt.% anthocyanin-coated) test paper were 538.6 eV and 529.1 eV, which may be attributed to the interaction of the Sb element with some organic substances during the preparation of the test paper. It is speculated that the O atom of the C=O bond in the PC extract had a lone pair of electrons that coordinated with the empty orbital of Sb (III). Therefore, the binding energy peak of 538.6 eV corresponds to the Sb3d_3/2_ orbital spin splitting of C=O-Sb [30]. After exposure to H_2_S gas, the binding energy peaks of 530.0 eV and 539.4 eV correspond to Sb (III). Combined with the S2p spectra, the binding energy peaks of 164.5 eV and 163.3 eV can be attributed to the S2p_1/2_ and S2p_3/2_ orbital spin splits of C-S, while the binding energy peaks of 162.2 eV and 161.0 eV correspond to Sb_2_S_3_ [31].

### 2.3. SEM Images of Test Paper

**Figure 8:**
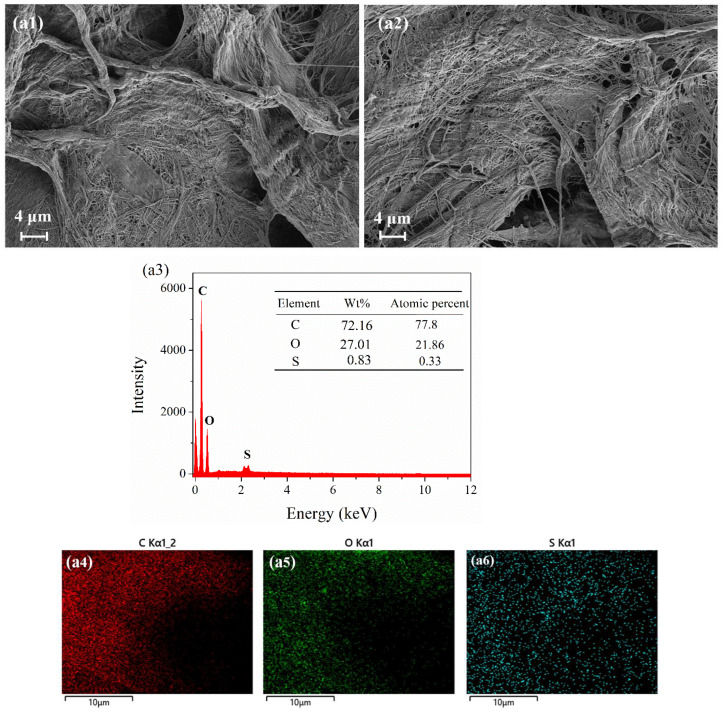

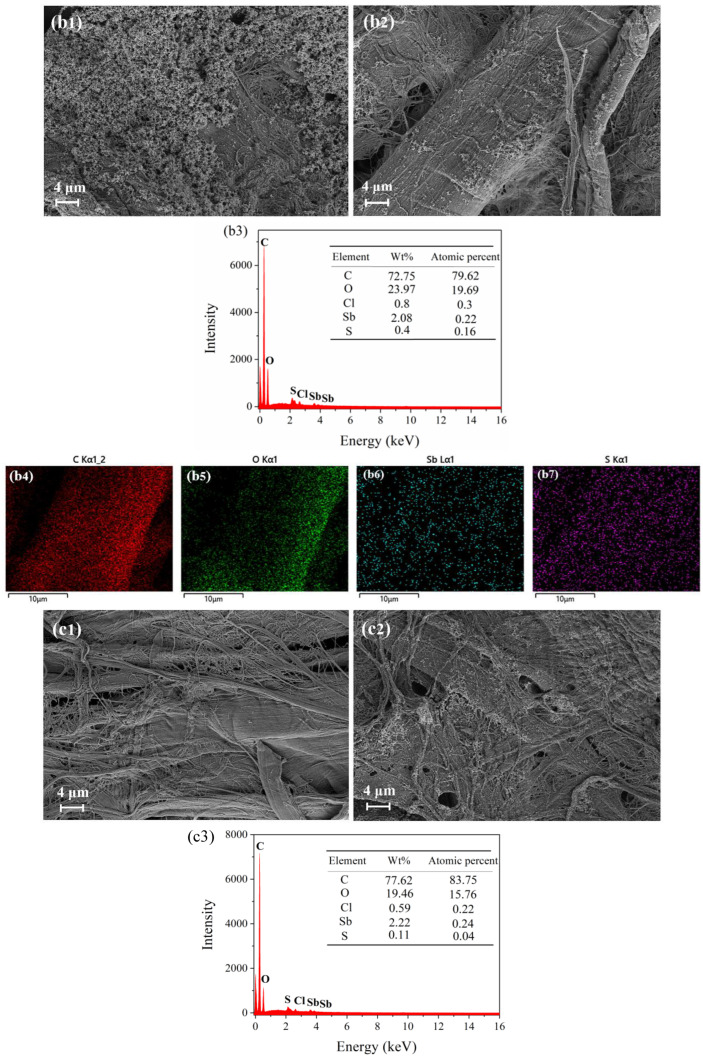

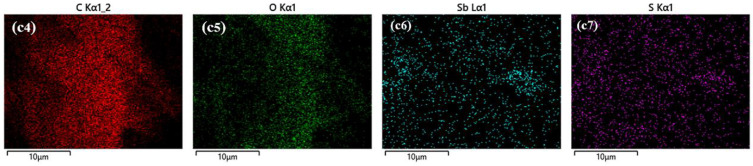
SEM images and EDX map of three test papers before and after being placed in 10 ppm H_2_S at 25 °C for 30 min. SEM images of 4 wt.% anthocyanin test paper (**a1**) before and (**a2**) after response and (**a3**–**a6**) EDS map after response. SEM images of 0.5 wt.% SbCl_3_ test paper (**b1**) before and (**b2**) after response and (**b3**–**b7**) EDS map after response. SEM images of 0.5 wt.% SbCl_3_ and 4 wt.% anthocyanin test paper (**c1**) before and (**c2**) after response and (**c3**–**c7**) EDS map after response.

Figure 8 shows the SEM images and EDX map of the different test papers before and after exposure to 10 ppm H_2_S for 30 min. Figure 8(a1,b1,c1) shows a large number of fibrous holes on the surface of the three test papers before exposure. These fibrous holes increased the contact area between the test paper and the H_2_S gas, making H_2_S adsorption easier. As shown in Figure 8(a1,a2), the test paper coated with 4 wt.% anthocyanin did not change significantly before and after exposure. However, the EDX analysis of the exposed surface showed that the atomic ratio of the S element was 0.33% (Figure 8(a3,a4)), indicating that the test paper coated with 4 wt.% anthocyanin could detect H_2_S gas, which is consistent with our previous research [20].

As shown in Figure 8(b1,b2), the test paper coated with 0.5 wt.% SbCl_3_ presented a uniform flocculent structure before exposure, which diminished after exposure. Combined with XPS, it was found that SbCl_3_ reacts with H_2_S gas to generate Sb_2_S_3_, but Sb_2_S_3_ has a poor binding force with the test paper and falls off slowly during the experiment. Similarly, the EDX analysis of the exposed test paper showed that the atomic ratio of the S element was 0.16% (Figure 8(b3–b5)), indicating that SbCl_3_ can effectively capture H_2_S gas.

Figure 8(c1,c2) show that the microstructure of the composite (0.5 wt.% SbCl_3_ and 4 wt.% anthocyanin-coated) test paper before exposure was similar to the 4 wt.% anthocyanin-coated test paper and after exposure, and it was identical to the 0.5 wt.%SbCl_3_-coated test paper. The S element was found in the EDX analysis of the exposed composite test paper. The contact with XPS indicated that SbCl_3_ and anthocyanin were bound in the process of test paper preparation. The combined SbCl_3_ was adsorbed on the test paper in the form of organic Sb. When exposed to H_2_S gas, some Sb elements reacted with H_2_S to form orange-yellow Sb_2_S_3_, which covered the surface of the test paper with a flocculent structure.

### 2.4. Mechanism Analysis

The reaction mechanisms of SbCl_3_-coated test paper and the SbCl_3_-and-anthocyanin-coated composite test paper were slightly different. Sb_2_S_3_ was generated by an inorganic reaction between the SbCl_3_-coated test paper and H_2_S, shown in Equation (1). Equation (2) shows the reaction mechanism of anthocyanins with H_2_S, which has been discussed in previous studies [20]. The mechanism of the composite test paper is that SbCl_3_ and anthocyanin are combined during its preparation. As shown in Equation (3), Sb (III) provides an empty orbital, and the O atom of the C=O bond in the PC extract feeds a lone pair of electrons that coordinate with the empty orbital of Sb (III). After exposure to H_2_S gas, SbCl_3_ and anthocyanin react with H_2_S to form Sb_2_S_3_ and C-S bonds, respectively.

SbCl_3_ + H_2_S → Sb_2_S_3_ + HCl (1)

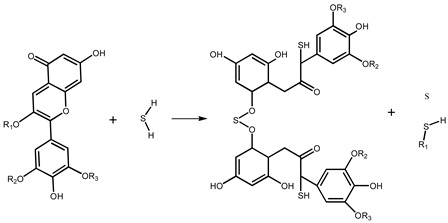
(2)

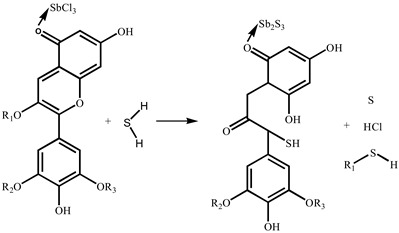
(3)

### 2.5. The Simulation of Pipeline Leakage

**Figure 9:**
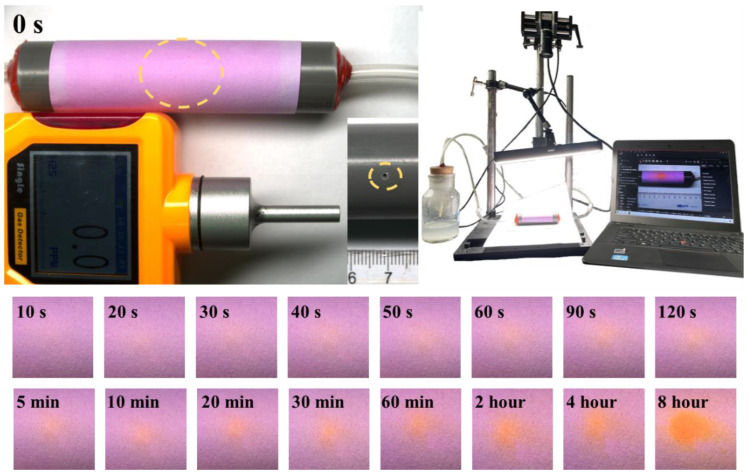
Schematic diagram of the device using test paper to monitor microleakage of pipeline and color change in test paper.

To validate the practical utility of our H_2_S test paper, we used it to test the discoloration of trace amounts of H_2_S gas after leakage from the pipeline (Figure 9 and Video S1, the video plays at triple speed). The transparent hose and the black plastic tube have small holes of 1 mm and the hose was placed inside the plastic tube, which was sealed with red glue at both ends. A known concentration of H_2_S gas (1000 ppm) generated by Na_2_S was pumped into the transparent hose through a circulation pump while the H_2_S sensor and video shooting software (HIKVISION) were turned on. The H_2_S sensor did not respond in any way during the experiment. After the experiment was completed, the time data of the color change were obtained by video capture. This result demonstrates that the H_2_S test paper prepared by us can detect microleakages that cannot be detected by ordinary instruments. At the same time, it has the characteristics of being cheap, convenient, and suitable for large-scale application, and is expected to provide a new H_2_S gas monitoring method for the field.

## 3. Materials and Methods

### 3.1. Experimental Material

The SbCl_3_, ethanol, and filter paper were purchased from Chengdu Kelon Chemical Reagent Factory (Chengdu, China). The anthocyanins were extracted from purple cabbage (PC), purchased from the local Carrefour.

### 3.2. Preparation of Response Test Paper

Different SbCl_3_ solutions were obtained by dissolving 0.005 g, 0.01 g, 0.02 g, 0.05 g, and 0.10 g of SbCl_3_ powder in 10 g of the ethanol–water mixture (1:1, wt/wt), respectively. The SbCl_3_ solutions (0.5 mL) were coated on different filter papers of the same size (2 cm × 5 cm). The filter papers were dried at room temperature (25 °C) and again coated with respective SbCl_3_ solution (0.5 mL). After drying, the response test papers coated with different concentrations of SbCl_3_ were obtained.

PC was dried in a vacuum oven (60 °C) for 36 h, then ground into powder for later use. Different weights of PC powder (1 g, 2 g, 3 g, 4 g, 5 g) were separately immersed in 50 mL ethanol–water (1:1, wt/wt) mixture, soaked for 2 h at room temperature, and then extracted using ultrasonic extractor for 30 min at 30 °C and 200 W. The extract was centrifuged at 3500 r/min and 10 °C for 15 min. The supernatant obtained was an anthocyanin solution. Mixed solutions of anthocyanin and SbCl_3_ were prepared by adding SbCl_3_ powder (0.05 wt.%, 0.1 wt.%, 0.2 wt.%, 0.5 wt.%, 1 wt.%, based on the weight of the anthocyanin solution) to different concentrations of anthocyanin solution and stirring for 10 min. Furthermore, the response test papers coated with mixed solutions were prepared similarly to SbCl_3_-coated test papers.

### 3.3. Visual Detection of H_2_S Gas

**Figure 10:**
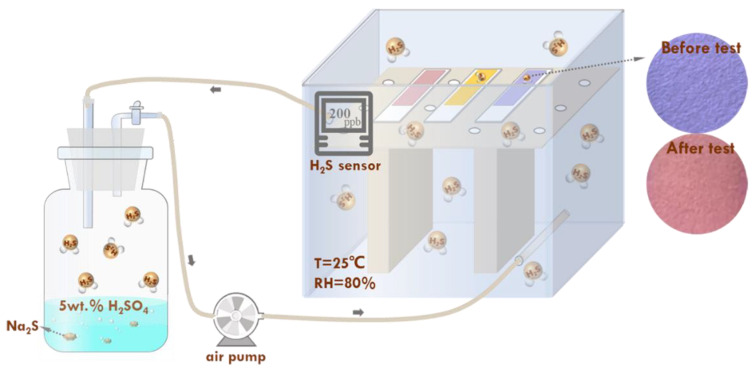
Schematic illustration of H_2_S response performance test process of the test paper.

As shown in Figure 10, the response test paper was placed in a glass container (on the right) to evaluate its responsiveness. A rubber hose and an air pump were used to connect the glass container to a 500 mL wide-mouth bottle. The glass container was filled with 100 mL of 5 wt.% H_2_SO_4_ solution and sealed with a rubber plug. Before the test, N_2_ was continuously introduced into the device to check the sealing performance. After the supply of N_2_ was stopped, solid Na_2_S·9H_2_O (mass of Na_2_S·9H_2_O corresponds to H_2_S gas concentration) was added to the H_2_SO_4_ solution to obtain the H_2_S gas. Simultaneously, the air pump was turned on to quickly fill the device with H_2_S gas. The H_2_S sensor in the glass container accurately indicated the H_2_S gas concentration. The change in color of the test paper was recorded using a colorimeter (YS4510 plus, 3nh, Shenzhen Sanenshi Technology Co., Ltd., Shenzhen, China) and a smartphone camera. The color difference value (∆*E*) was calculated using Equation (4) to digitize the color change in the test paper [17]:

(4) $$∆E=\sqrt{{\left(L^{*}-L_{0}^{*}\right)}^{2}+{\left(a^{*}-a_{0}^{*}\right)}^{2}+{\left(b^{*}-b_{0}^{*}\right)}^{2}}$$

where $L_{0}^{*}$ (luminance), $a_{0}^{*}$ (red-green value), and $b_{0}^{*}$ (yellow-blue value) were the initial parameters, and L*, a*, and b* were the parameters obtained after H_2_S gas exposure.

Additionally, the environmental influences on the response test paper were investigated by varying the experimental temperature (0 °C, 25 °C, 60 °C, 80 °C) and introducing other gases (N_2_, CO_2_, O_2_, air) into the device. The H_2_S detection was investigated using different test papers coated with different concentrations of SbCl_3_ and/or anthocyanin.

### 3.4. Characterization Methods

A scanning electron microscope (SEM, EVOMA15, ZEISS, Aalen) was used to examine the microscopic morphology of the test papers before and after the introduction of H_2_S gas. Before observing under SEM, the surface of the test papers was coated with gold nanoparticles. The surface element content was analyzed using energy-dispersive X-ray spectroscopy (EDX). Furthermore, the surface composition and chemical valences were analyzed using X-ray photoelectron spectroscopy (XPS, Thermo Scientific K-Alpha, USA).

## 4. Conclusions

In summary, a portable and efficient colorimetric test paper was developed using SbCl_3_ and anthocyanin to detect H_2_S gas quantitatively. It can be used to monitor the leakage of H_2_S gas by color change that is visual to the naked eye and can determine the leakage concentration of H_2_S gas when used in conjunction with a colorimeter. The H_2_S detection limit of the test paper prepared in this work is 200 ppb, which is significantly lower than the existing test papers for H_2_S detection. Moreover, the test paper is not sensitive to common gases in the air such as O_2_, N_2_, and CO_2_, demonstrating that it is not affected by other gases and can be stored for a long time. Overall, the selectivity of this test paper to H_2_S provides a new strategy for the rapid detection and quantification of H_2_S gas leakage in industries.

Under the background of the gradual development of artificial intelligence, the robot is expected to recognize and digitize the color change during the patrol process and implement an automatic alarm when the H_2_S concentration reaches the dangerous range.

## Data Availability

All raw/processed data necessary for reproducing the results in this study can be accessed upon reasonable request.

## Supplementary Materials

The following supporting information can be downloaded at https://www.mdpi.com/article/10.3390/molecules28135044/s1: Video S1: Three times the speed of the simulated pipeline leak experiment.

## Sample Availability

The synthesized test paper samples are available from the authors.
