# Supplementary material for: A Visual Color Response Test Paper for the Detection of Hydrogen Sulfide Gas in the Air
Source: Molecules. 2023 Jun 28;28(13):5044. doi: 10.3390/molecules28135044 (PMC10343175; doi:10.3390/molecules28135044)
Supplement: Supplementary file 1 [file molecules-28-05044-s001.zip › Supporting Information.pdf]

Supporting Information

A visual color response test paper for the hydrogen sulfide gas monitoring in the air

Hailong Zhang[1,4], Shiyu Li[1], Zhongzhi Han[5], Hongpeng Zheng[1], Junlei Tang[1,2*], Bing Lin[1], Yingying Wang[3], Xiaojun Guo[5], Taigang Zhou[1,2], Jianjun Wu[4], Haibing Zhang[6], Hui Zhang[1]

[1] *School of Chemistry and Chemical Engineering & Institute for Carbon Neutrality, Southwest Petroleum University, Chengdu 610500, China.*

[2] *Tianfu Yongxing Laboratory, Chengdu 610217, China.*

[3] *Key Laboratory of Optoelectronic Chemical Materials and Devices (Ministry of Education), Jianghan University, Wuhan 430056, China.*

[4] *Research Institute of Tianfu New Energy, Chengdu 610217, China.*

[5] *CNPC Engineering Technology Research Company Limited, Tianjin 300451, China.*

[6] *State Key Laboratory for Marine Corrosion and Protection, Luoyang Ship Material Research Institute, Qingdao 266237, China.*

**Corresponding authors:*

Name: Junlei Tang, E-mail: tangjunlei@126.com

Name: Shiyu Li, E-mail: 1198431819@qq.com

Supporting Information: triple speed.mp4

Video S1 Three times the speed of the simulated pipeline leak experiment
